# Contrast-enhanced ultrasound for the preoperative prediction of pathological characteristics in breast cancer

**DOI:** 10.3389/fonc.2024.1320714

**Published:** 2024-02-29

**Authors:** Ling-Ling Li, Quan-Li Su, Yun-Xia Deng, Wen-Wen Guo, Hai-Mei Lun, Qiao Hu

**Affiliations:** ^1^ Departments of Ultrasound, The People’s Hospital of Guangxi Zhuang Autonomous Region, Nanning, Guangxi, China; ^2^ Departments of Pathology, The People’s Hospital of Guangxi Zhuang Autonomous Region, Nanning, Guangxi, China

**Keywords:** contrast-enhanced ultrasound, VueBox, breast cancer, histological grade, molecular subtypes

## Abstract

**Objective:**

We aimed to investigate the value of contrast-enhanced ultrasound (CEUS) in the preoperative prediction of the histological grades and molecular subtypes of breast cancer.

**Methods:**

A total of 183 patients with pathologically confirmed breast cancer were included. Contrast enhancement patterns and quantitative parameters were compared in different groups. The receiver operating characteristic (ROC) curve was used to analyze the efficacy of CEUS in the preoperative prediction of pathological characteristics, including histologic grade and molecular subtypes.

**Results:**

Heterogeneous enhancement, perfusion defects, and peripheral radial vessels were mostly observed in higher histologic grade (grade III) breast cancer. Heterogeneous enhancement and perfusion defect were the most effective indicators for grade III breast cancer, with the areas under the ROC curve of 0.768 and 0.756, respectively. There were significant differences in the enhancement intensity, post-enhanced margin, perfusion defects, and peripheral radial vessel among the different molecular subtypes of breast cancer (all *P* < 0.01). Perfusion defects and clear edge after enhancement were the best qualitative criteria for the diagnosis of HER-2 overexpressed and triple-negative breast cancers, and the corresponding areas under the ROC curves were 0.804 and 0.905, respectively. There were significant differences in PE, WiR, WiPI, and WiWoAUC between grade III vs grade I and II breast cancer (*P* < 0.05). PE, WiR, WiPI, and WiWoAUC had good efficiency in the diagnosis of high-histologic-grade breast cancer. PE had the highest diagnostic efficiency in Luminal A, while WiPI had the highest diagnostic efficiency in Luminal B subtype breast cancer, and the areas under the ROC curve were 0.825 and 0.838, respectively. WiWoAUC and WiR were the most accurate parameters for assessing triple-negative subtype breast cancers, and the areas under the curve were 0.932 and 0.922, respectively.

**Conclusion:**

Qualitative and quantitative perfusion analysis of contrast-enhanced ultrasound may be useful in the non-invasive prediction of the histological grade and molecular subtypes of breast cancers.

## Introduction

According to the latest analysis from the International Agency for Research on Cancer, new cases of breast cancer surpassed those of lung cancer in 2020, becoming the world’s most commonly diagnosed cancer (1). Breast cancer is a highly heterogeneous tumor, and the pathological grading of breast cancer reflects the degree of malignancy and invasiveness of the tumor to a certain extent (2). The higher the pathological grading, the more malignant the tumor, and the worse the prognosis. The expression levels of estrogen receptor (ER), progesterone receptor (PR), and human epidermal growth factor receptor 2 (HER2) in breast cancer cells are considered important factors in determining the biological behavior of breast cancer and the efficacy of endocrine therapy and are the main prognosis predictors (2, 3). For the preoperative assessment of the histopathological grading and molecular subtyping of breast cancer, the clinical standard is the pathological immunohistochemistry of needle biopsies. However, pathological biopsy is an invasive procedure and has the defect of insufficient samples to make a diagnosis. Therefore, it is necessary to develop non-invasive imaging modalities to predict the pathological characteristics of breast cancer before surgery.

VueBox is a color-coded external perfusion software program that can be used for dynamic contrast-enhanced ultrasound (CEUS) with motion/respiration compensation and is suitable for DICOM format video images acquired by various ultrasound equipment (4). The software calculates the perfusion parameters automatically, generating color-coded maps of the perfusion parameters and thus providing a more direct and objective quantitative analysis of the subtle difference in enhancement degree, reducing the subjective dependence of image interpretations by operators. In recent years, there have been literature reports on the application of VueBox to quantitatively analyze the characteristics of breast, thyroid, liver, and other organ tumors by CEUS (5–7). Jung EM (7) et al. used the TIC curve of VueBox external perfusion software to compare and analyze the perfusion performance of benign and malignant non-cystic breast masses. The results showed that the quantitative CEUS perfusion parameters PE and areas under the curve (AUC) can well evaluate the malignant risk of non-cystic breast masses. This may reduce the risk rating for certain BI-RADS category 4 lesions. However, there are few reports on the application of CEUS perfusion imaging with VueBox for the evaluation of breast cancer pathological characteristics. In this study, VueBox external perfusion analysis software was used to explore the value of contrast-enhanced ultrasound (CEUS) in the preoperative prediction of the pathological grading and molecular subtyping of breast cancer.

## Materials and methods

### Patients

This study was performed with the approval of the Ethics Committee of the People’s Hospital of Guangxi Zhuang Autonomous Region, China (IRB No. KY-LW-2020-24). Informed consent was obtained from all participants. A total of 183 patients with breast cancer were enrolled from December 2020 to April 2023 (women, age range 28-85 y, and mean age 52 y). Inclusion criteria: ① patients who underwent CEUS and for whom contrast dynamic images were stored; ② no preoperative treatment; ③ pathologically confirmed invasive breast cancer; and ④ complete postoperative pathological and immunohistochemical results. The exclusion criteria were as follows: ① poor quality of CEUS dynamic images; and ② incomplete clinical data.

### Imaging acquisition

Conventional ultrasound and CEUS imaging were performed using a GE LOGIQ E9 ultrasound system (GE Healthcare, Chicago, IL, USA) with a high-resolution linear transducer; the probe frequency was 6-15 MHz for conventional ultrasound and 6-9 MHz for CEUS. The patients were placed in a supine position with arms placed above the head. First, the whole breast was continuously multi-section scanned by conventional ultrasound. Color and power Doppler were performed in different planes to evaluate the intralesional vascularity. Then, the plane with the most abundant vessels including the lesion and its surrounding normal breast tissue was selected and switched to CEUS imaging mode with a mechanical index (MI) < 0.10. The ultrasound contrast agent used in the present study was SonoVue (Bracco SpA, Milan, Italy). A bolus of 4.8 mL of contrast agent was administrated via a peripheral vein and was immediately followed by a flush of 5 mL saline. CEUS continuous dynamic imaging was observed immediately after injection of the contrast agent for at least 6 minutes. The images and video clips (the last 2 minutes after contrast agent injection) were stored and transferred to a mobile hard disk in Dicom format for subsequent offline analysis.

### CEUS image analysis

CEUS image analysis was performed by two radiologists (LL.L and Q.H with 3 and 12 years of experience in breast CEUS, respectively). They were blinded to the clinical data and pathological results of the patients. In cases of discrepancies, the two reviewers reanalyzed and discussed together to reach a consensus.

The following CEUS qualitative indicators were analyzed: (1) compared with that of surrounding normal breast tissue, the enhancement intensity was classified as hyper-, iso-, or hypo-enhancement; (2) based on the internal homogeneity of the tumor, enhancement was divided into homogeneous or heterogeneous enhancement; (3) the enhancement edge of the lesion was classified as a clear or blurred margin; (4) whether the lesion scope enlarged after enhancement; (5) the presence or absence of perfusion defect; (6) the presence or absence of radial or penetrating vessels.

Quantitative analysis for dynamic CEUS imaging was performed using the color-coded off-line software (VueBox, Bracco, Genève, Suisse). Regions of interest were manually delineated with the strongest enhanced area in the lesion and the surrounding normal breast tissue at the same depth. Time-intensity curves were generated to obtain quantitative parameters, including peak enhancement (PE), mean transit time (mTT), time to peak (TTP), wash-in rate (WiR), wash-in perfusion index (WiPI), and wash-in and wash-out areas under the curve (WiWoAUC). For each quantitative data, the measurements were repeated three times, and their mean was used in the analysis.

### Histopathologic analysis

Histopathological specimens were fixed with 10% formalin, embedded in paraffin, and sliced into 3-μm sections, and hematoxylin–eosin staining was performed. Invasive breast cancer was graded using the Nottingham histological grading system (8). Immunohistochemical staining was used to determine the expressions of estrogen receptor (ER), progesterone receptor (PR), human epidermal growth factor receptor 2 (Her-2), and Ki-67. The molecular classification of breast cancer was divided into four subtypes (9): ① luminal A subtype: ER(+) and/or PR(+), Her-2(-), and Ki-67 low expression (< 14%); ② luminal B subtype: ER(+) and/or PR(+), Her-2(+), or ER(+) and/or PR(+), Her-2(-), and Ki-67 high expression (≥ 14%); ③ Her-2 overexpressed subtype: ER(-), PR(-), and Her-2(+); and ④triple-negative subtype: ER(-), PR(-), and HER-2(-). All histopathologic slides were observed by a pathologist (WW.G) who had more than 6 years of experience in breast pathologic analysis.

### Statistical analysis

SPSS 26.0 statistical software (IBM Corp, Armonk, NY, USA) was used for the statistical analysis. Count data are expressed as frequency (n), and intergroup comparisons were conducted using the χ² test. Quantitative parameters with a non-normal distribution were presented as M (Q1, Q3); the Kruskal−Wallis H test was used for intergroup comparisons of quantitative data, and the Dunn-Bonferroni test was used for pairwise comparisons. MedCalc software was also used to draw ROC curves for the parameters with significant differences to verify their diagnostic efficacy. *P* < 0.05 was considered statistically significant.

## Results

There were 183 invasive breast cancers among 183 patients enrolled in this present study, including invasive ductal carcinoma (n = 152, 83.06%), invasive lobular carcinoma (n = 20, 10.93%), intraductal papillary carcinoma (n = 3, 1.64%), mucinous carcinoma (n = 3, 1.64%), and medullary carcinoma (n = 5, 2.73%). Of all the breast lesions (size, 28.8 ± 19.2 mm, range, 8.0-118mm), 126 (68.85%) were moderately (grade II) and highly differentiated (grade I), 57 (31.15%) were poorly differentiated (grade III), 50 (27.32%) were luminal A, 80 (43.72%) were luminal B, 31 (16.94%) were HER-2 overexpressed, and 22 (12.02%) were triple-negative subtypes. There were no significant differences in age and tumor size among patients with different grades and subtypes (**Table 1**).

**Table 1:** Clinicopathological Characteristics of patients.

Characteristics	Case number (percentage)
Age (years)*	52.21 ± 11.54
Tumor diameter (mm)*	28.82 ± 19.24
Histologic grade	
Grades I and II	126 (68.85%)
Grade III	57 (31.15%)
Molecular subtypes	
Luminal A	50 (27.32%)
Luminal B	80 (43.72%)
Her-2 overexpressed	31 (16.94%)
Triple-negative	22 (12.02%)
Histologic type	
Invasive ductal carcinoma	152 (83.06%)
Invasive lobular carcinoma	20 (10.93%)
Intraductal papillary carcinoma	3 (1.64%),
Mucinous carcinoma	3 (1.64%)
Medullary carcinoma	5 (2.73%)
Lymph node status	
Positive	60 (32.79%)
Negative	123 (67.21%)

* Mean ± standard deviation.

### Qualitative CEUS features of breast cancer with different pathological grades and molecular subtypes

**Figure 1:**
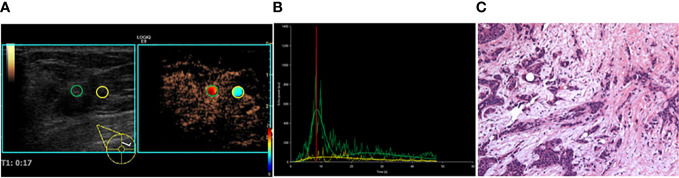
A 35-year-old female patient with invasive ductal carcinoma. **(A)** CEUS perfusion imaging using VueBox software. The ROIs were set in the breast lesion (green circle) and the surrounding breast tissue (yellow circle). The breast lesion showed iso-hyper, homogeneous enhancement, and no perfusion defect, and the radial vessel was observed. **(B)** TIC analysis showed a fast wash-in, a medium-high peak, and a rapid wash-out with the tumor. **(C)** Histopathological examination indicated a moderately differentiated (grade II), Luminal B subtyping breast cancer (HE, ×100). TIC, Time intensity curve.

The qualitative CEUS analysis revealed that higher histological grade (grade III) breast cancer mostly showed heterogeneous enhancement (50/57, 87.72%), perfusion defect (41/57, 71.93%), and presence of radial or penetrating vessels (47/57, 82.46%). Lower histological grade (grade I and II) breast cancer showed more iso- or hypo-enhancement (79/126, 62.70%), homogeneous enhancement (83/126, 65.87%), and no obvious perfusion defect (100/126, 79.37%) (**Figure 1**). The enhancement degree, internal homogeneity, perfusion defect, and presence or absence of radial or penetrating vessels showed significant differences between lower histological grade and higher histological breast cancer (all *P* <0.01). However, with regard to the enhancement edge and whether lesion scope enlarged after enhancement, no statistical difference was found between the two groups (*P* > 0.05) (**Table 2**).

**Table 2:** CEUS Qualitative Features with Different Histologic grades of Breast Cancer.

Histologic grade	Enhancement Intensity		Internal Homogeneity		Enhancement edge		Perfusion defect		Radial or penetrating vessel		Enhancement scope enlarged	
	Hyper-enhancement	Iso- or hypo-enhancement	Homogeneous	Heterogeneous	clear	blurred	Present	Absent	Present	Absent	Present	Absent
Grades I and II	47	79	83	43	24	102	26	100	45	81	77	49
Grade III	44	13	7	50	12	45	41	16	47	10	42	15
Total	91	92	90	93	36	147	67	116	92	91	119	64
*P* Value	< 0.01		< 0.01		0.752		< 0.01		< 0.01		0.099	

**Figure 2:**
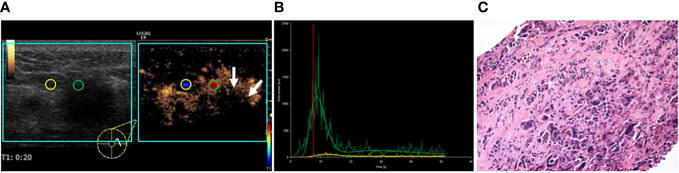
A 55-year-old female patient with invasive ductal carcinoma. **(A)** VueBox perfusion imaging showed that hyper-, heterogeneous enhancement, and perfusion defect (white arrows) were present in the breast lesion. **(B)** TIC analysis showed a fast wash-in, a higher peak, and a rapid wash-out with the tumor. **(C)** Histopathological examination indicated a poorly differentiated (grade III), Her-2 subtyping breast cancer (HE, ×100). TIC: Time intensity curve.

**Figure 3:**
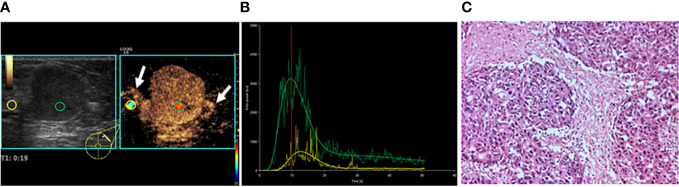
A 47-year-old female patient with invasive lobular carcinoma. **(A)** CEUS perfusion imaging showed that hyper-enhancement, clear edge, and radial vessels (white arrows) were present in the breast lesion. **(B)** TIC analysis showed a fast wash-in, a higher peak, and a rapid wash-out with a greater WiWoAUC of the tumor. **(C)** Histopathological examination indicated a poorly differentiated (grade III), triple-negative subtyping breast cancer (HE, ×100). WiWoAUC: wash-in and wash-out area under the curve.

Iso- or hypo-enhancement was found in 43 (43/50, 86.00%) luminal A and 41 (41/80, 51.25%) luminal B subtype breast cancers. There were 44 (44/50, 88.00%) luminal A and 42 (42/80, 52.25%) luminal B lesions present in the radial or penetrating vessels. The enhancement features of HER-2 breast cancer were predominantly hyper-enhancement (26/31, 83.87%) and perfusion defects (27/31, 87.10%). The enhancement features of triple-negative breast cancer were predominantly clear edge (20/22, 90.91%) (**Figures 2**, **3**). There were significant differences in the enhancement degree, enhancement edge, perfusion defects, and radial or penetrating vessels among the different molecular subtypes of breast cancer (*P* < 0.05). Radial or penetrating vessels were more common in luminal A breast cancer than in other subtypes. In addition, clear edge after enhancement was more common in the triple-negative subtype, and perfusion defect was more often found in HER-2 overexpressed breast cancer than in other subtypes (*P* < 0.05). With regard to internal homogeneity, there was no significant difference among the different molecular subtypes (*P* > 0.05) (**Table 3**).

**Table 3:** CEUS Qualitative Features with Different Molecular Subtypes of Breast Cancer.

Molecular Subtype	Enhancement Intensity		Internal Homogeneity		Enhancement edge		Perfusion defect		Radial or penetrating vessel		Enhancement scope enlarged	
	Hyper-enhancement	Iso- or hypo-enhancement	homogeneous	Heterogeneous	clear	blurred	Present	Absent	Present	Absent	Present	Absent
Luminal A	7	43	23	27	6	44	15	35	44	6	32	18
Luminal B	39	41^a^	35	45	5	75	23	57	42	38^a^	54	26
Her-2 overexpressed	26	5^ab^	18	13	5	26	27^ab^	4	4	27^ab^	19	12
Triple-negative	19	3^ab^	14	8	20^abc^	2	2	20^c^	2	20^ab^	14	8
Total	91	92	90	93	36	147	67	116	92	91	119	64
*P* Value	< 0.01		0.262		< 0.01		< 0.01		< 0.01		0.930	

a Compared with the Luminal A subtype, p < 0.05.

b Compared with the Luminal B subtype, p < 0.05.

c Compared with the Her-2 overexpressed subtype, p < 0.05.

### Quantitative CEUS parameters of breast cancer with different pathological grades and molecular subtypes

There were significant differences in PE, mTT, TTP, WiR, WiPI, and WiWoAUC between breast cancer groups and the surrounding normal breast tissue (all *P* < 0.001). After Bonferroni correction, the PE, WiR, WiPI, and WiWoAUC for breast cancer lesions were greater, while mTT and TTP were shorter than the surrounding normal breast tissue. PE, WiR, WiPI, and WiWoAUC for the higher histological grade group were greater than the lower histological grade group (**Table 4**).

**Table 4:** Comparison of CEUS Quantitative Parameters in different histological grades of breast cancer and normal breast tissue *(Q1, Q3)*.

Group	PE (au)	mTT (s)	TTP (s)	WiR (au)	WiPI (au)	WiWoAUC (au)
Normal breast tissue	95.72 (39.19, 295.39)	39.63 (21.70, 73.87)	12.29 (8.98, 17.32)	17.67 (7.57, 51.12)	69.69 (27.06, 402.93)	1101.93 (341.01, 3869.24)
Grades I and II	840.19^a^ (414.84, 2351.48)	26.56^a^ (18.19, 52.72)	8.25^a^ (6.57, 9.84)	151.26^a^ (78.28, 269.91)	726.95^a^ (277.77, 4146.50)	7400.29^a^ (2672.86, 19547.71)
Grade III	2926.97^ab^ (1015.94, 14848.96)	22.58^a^ (14.04, 44.52)	7.74^a^ (6.57, 9.84)	604.97^ab^ (191.32, 3013.64)	2107.34^ab^ (727.08, 9465.63)	26677.88^ab^ (9280.65, 146525.00)
*P* Value	< 0.001	< 0.001	< 0.001	< 0.001	< 0.001	< 0.001

^a^Compared with normal breast tissue, p < 0.05; ^b^Compared with Grades I and II breast cancer, p < 0.05.

PE, peak enhancement; mTT, mean Transit time; TTP, Time to Peak; WiR, wash-in rate; WiPI, wash-in perfusion index; WiWoAUC, wash-in and wash-out areas under the curve.

au, arbitrary unit; S, second.

There were significant differences in the quantitative parameters PE, WiR, WiPI, and WiWoAUC among the different molecular subtypes of breast cancer (all *P* < 0.05). PE, WiR, WiPI, and WiWoAUC for the luminal A and luminal B subtypes were lower than triple-negative breast cancer. HER-2 overexpressed subtype had higher PE than luminal A and greater WiR than luminal A and luminal B subtypes. However, the mTT and TTP showed no statistical difference among the different molecular subtypes of breast cancer (both *P* > 0.05) (**Table 5**).

**Table 5:** CEUS Quantitative Parameters with Different Molecular Subtypes of Breast Cancer *(Q1, Q3)*.

Molecular Subtype	PE (au)	mTT (s)	TTP (s)	WiR (au)	WiPI (au)	WiWoAUC (au)
Luminal A	710.56 (333.10, 2584.27)	23.41 (18.04, 51.69)	8.70 (7.23, 10.04)	115.97 (62.10, 203.20)	833.74 (241.40,4106.77)	7617.21 (2104.91,38584.92)
Luminal B	876.70 (442.76, 3357.51)	23.56 (16.87, 44.9175)	7.975 (6.98, 10.3625)	175.97 (83.92, 427.59) ^a^	817.85 (325.13,4424.32)	10368.69 (3927.83,45378.18)
Her-2 overexpressed	2082.53 (729.85, 6682.69)^a^	29.00 (15.11, 54.04)	8.36 (7.28, 10.72)	629.8 (197.28,1361.85)^ab^	1762.22 (465.52,22005.59)	12184.65 (4372.72,36945.39)
Triple-negative	2946.19 (1565.76, 16195.12)^ab^	29.495 (14.43, 52.09)	7.74 (6.40, 9.62)	879.26 (230.51,5049.16)^ab^	2116.12 (1132.53,13369.86)^a,b^	34138.295 (11401.22,168665.78)^a,b^
*P* Value	< 0.001	0.842	0.478	< 0.001	< 0.001	0.007

^a^Compared with Luminal A subtype, p < 0.05; ^b^Compared with Luminal B subtype, p < 0.05.

PE, peak enhancement; mTT, mean Transit time; TTP, Time to Peak; WiR, wash-in rate; WiPI, wash-in perfusion index; WiWoAUC, wash-in and wash-out areas under the curve.

au, arbitrary unit; S, second.

### Diagnostic performances of different qualitative and quantitative CEUS parameters

**Figure 4:**
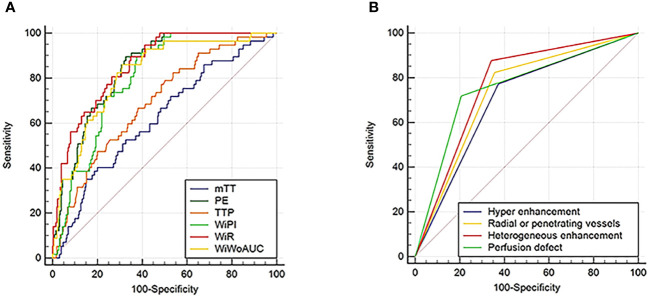
ROC curves for CEUS quantitative **(A)** and qualitative **(B)** parameters in the diagnosis of higher histologic grade (grade III) breast cancer. ROC, Receiver operating characteristic; CEUS, Contrast enhanced ultrasound.

Using the pathological results as the gold standard, ROC curves were drawn to analyze the diagnostic performance of CEUS perfusion imaging with VueBox for the pathological grades and molecular subtypes of breast cancer. The results showed that among the qualitative parameters, perfusion defect and heterogeneous enhancement were the most accurate features for higher histologic-grade breast cancer, and the areas under the ROC curve were 0.756 and 0.768, respectively. Among the quantitative parameters, PE, WiR, and WiWoAUC had the highest diagnostic performance, with 667.02, 108.55, and 6517.99 identified as the optimal cutoff values for the diagnosis of higher histologic grade breast cancer; the corresponding sensitivities were 0.895, 0.895, and 0.860; specificities were 0.673, 0.651, and 0.689; and the accuracies were 0.708, 0.689, and 0.716, respectively (**Figure 4**).

**Figure 5:**
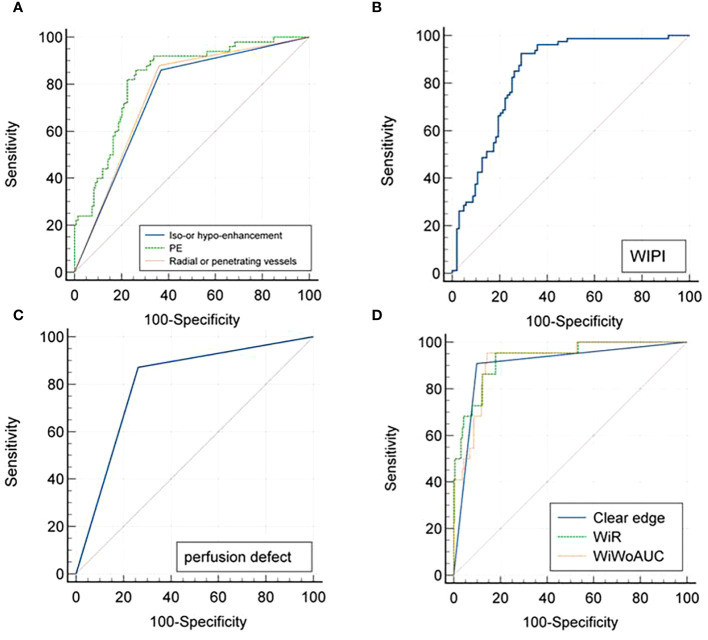
ROC curves for CEUS diagnoses of **(A)** Luminal A, **(B)** Luminal B, **(C)** Her-2 overexpressed, and **(D)** Triple-negative subtyping breast cancer. ROC, Receiver operating characteristic; CEUS, Contrast-enhanced ultrasound.

With a PE value of 253.96 as the threshold, hypo-enhancement, and the presence of radial or penetrating vessels used to diagnose luminal A breast cancer, the areas under the ROC curve were 0.825, 0.746, and 0.760, respectively. Using the cutoff value of 147.56 for WiPI to diagnose luminal B subtype breast cancer, the area under the ROC curve was 0.838, and the sensitivity, specificity, and accuracy were 0.925, 0.709, and 0.803. Perfusion defect and clear edge after enhancement were the best qualitative criteria for diagnosis of HER-2 overexpress and triple-negative breast cancer; the corresponding areas under the ROC curves were 0.804 and 0.905, the corresponding sensitivities were 0.871 and 0.909, specificities were 0.737 and 0.901, and the accuracies were 0.760 and 0.902, respectively. Using a WiR value of 107.81 and WiWoAUC value of 7646.07 as the cutoff values to diagnose triple-negative breast cancer, the areas under the curve were 0.932 and 0.922, the sensitivities were 0.955 and 0.955, the specificities were 0.820 and 0.857, and the accuracies were 0.836 and 0.869, respectively (**Figure 5**).

## Discussion

Previous studies have reported that CEUS enhancement patterns and hemodynamic changes in breast cancer can be analyzed to predict the pathological characteristics associated with breast cancer prognosis (10). However, previous studies on the preoperative assessment of breast cancer pathological grades and ER, PR, or other biomarkers’ expression by CEUS mostly focused on qualitative indicators, which have certain limitations of subjectivity. In the present study, VueBox, an external perfusion analysis software, was used to comprehensively analyze and explore the value of CEUS qualitative and quantitative parameters in the preoperative assessment of the pathological grading and molecular classification of breast cancer.

The results indicated that higher histological-grade breast cancer commonly showed heterogeneous enhancement, perfusion defects, and the presence of radial or penetrating vessels. Additionally, iso- or hypo-enhancement, homogeneous enhancement, and no obvious perfusion defect were found more often in lower histological-grade breast cancer. These findings are consistent with the results of a previous report (11). Breast cancer is a vascular-dependent disease, and the differences in CEUS enhancement patterns between breast cancer lesions and the surrounding normal breast tissue are closely related to their blood perfusion and pathological characteristics. The higher the histological grade of breast cancer, the poorer the differentiation, the higher the degree of malignancy, and the more angiogenesis (12). The distribution of blood vessels in malignant tumors is uneven; there are abundant tortuous and dilated blood vessels at the edges of lesions, and immature, stenotic, and occluded new blood vessels are common, resulting in heterogeneous enhancement within the tumors (13). Perfusion defects in malignant lesions are related to rapid tumor growth and the relatively insufficient supply of oxygen and nutrients, resulting in tumor liquefaction and necrosis. On CEUS, radial or penetrating vessels may manifest as the “crab claw” sign. As we know, cells of tumors with higher histological grades could continuously secrete a large amount of vascular endothelial growth factor, which promotes the formation of new blood vessels and infiltration into surrounding normal tissue. These tumors are likely to appear as “crab claw”-like enhancement on CEUS (14).

To minimize the influence of subjective factors and individual differences on the interpretation of CEUS imaging, VueBox quantitative analysis was also used to analyze the differences in breast cancer and surrounding normal breast tissue. The results showed that the TIC curve for breast cancer lesions was characterized by rapid and hyper-perfusion. Quantitative CEUS parameters for breast cancer lesions were significantly different from those for surrounding normal breast tissue. It is probably due to the differences between the microvessel density (MVD) of the lesions and the surrounding normal glandular tissue. There is very little neovascularization in normal breast tissue, and the MVD in breast cancer lesions is significantly higher than that in normal breast tissue (15). Additionally, higher histological-grade breast cancer has more thick feeding vessels, and the neovascular wall in the tumor is incomplete. A lack of smooth muscle innervation and vasomotor components and the formation of thrombi in feeding vessels in the tumor can cause a large number of microbubbles to remain in the blood vessels and eventually lead to greater PE, WiR, WiPI, and WiWoAUC and a shorter mTT and TTP values for the breast cancer lesion than for the surrounding normal glandular tissue (16). However, Li et al. (13) proposed that the longer the TTP, the smaller the WiR, and the higher the pathological grade, which is different from the present finding. The explanation for this inconsistent result may be attributable to the individual differences of patients, different ROI areas, or analysis software (17).

In this study, luminal A and luminal B subtypes of breast cancer mostly showed iso- or hypo-enhancement on CEUS. The reason for this may be the low density of microvessels, low invasiveness, and low perfusion in luminal epithelial tumors. Radial or penetrating vessels on CEUS are characteristic of luminal A breast cancer. It is speculated that luminal A breast cancer has lower Ki-67 expression, slower cell proliferation, and lower malignancy. In addition, tumor adhesion and E-cadherin expression promote the proliferation of interstitial connective tissue and inflammatory cell infiltration, leading to the formation of dense fibrosis. On CEUS, these tumors appear peripherally radial convergent, which is consistent with the burr-like appearance around masses on mammography (18).

HER-2 expression is correlated with tumor size, lymph node metastasis, and TNM stage. HER-2 overexpression often indicates poor prognosis (19). In our present study, HER-2 breast cancer mostly showed hyper-enhancement and perfusion defects. HER-2 can upregulate the expression of vascular endothelial growth factor (VEGF), increase angiogenesis, and stimulate the proliferation of microvessels around a tumor (20). The blood supply to the tumor increases, manifesting as hyper-enhancement on contrast-enhanced ultrasonography. When a tumor grows rapidly, necrosis occurs due to insufficient oxygen and nutrient supply, manifesting as perfusion defects on CEUS, which is consistent with the results reported by Liang et al. (21).

Triple-negative breast cancer has the worst prognosis and is not sensitive to endocrine therapy and targeted therapy. Previous studies have indicated that triple-negative breast cancer and benign tumors have similar appearances on conventional ultrasound (22). In this study, triple-negative breast cancer commonly showed a clear edge on CEUS, potentially relating to the compressive growth of triple-negative breast lesions, and the stromal reaction around the gland is reduced, resulting in a clear border between the tumor and the surrounding breast tissue (23).

There were different opinions of previous research regarding the correlation between quantitative CEUS parameters and the molecular expression in breast cancer. Vraka et al. (10) reported that there was no significant difference in PE, TTP, and MTT between ER-negative and ER-positive tumors. The results of another study suggested that the PE of luminal epithelial breast cancer was lower than that of HER-2 and triple-negative tumors, while the TTP of HER-2 breast cancer was shorter than that of other subtypes (21). Our results revealed that PE, WiR, WiPI, and WiWoAUC for the luminal A and luminal B subtypes were lower than triple-negative breast cancer. HER-2 overexpressed subtype had higher PE than luminal A and greater WiR than luminal A and luminal B subtypes. Our results concur with those of Wen B et al. (24) in that HER-2 overexpressed and triple-negative breast lesions can secrete more vascular endothelial growth factor, leading to higher angiogenesis and vascular permeability and significant hyper-perfusion situation in the tumors.

This study had certain limitations. First, this was a retrospective study, and there may be some selection bias. Second, the selected ROIs were lesion areas with the strongest enhanced area, thus not fully representing blood perfusion in entire lesions. Third, univariate analysis was used in the present study and the sample size was relatively small; the combined value of CEUS quantitative and qualitative parameters in predicting the pathological characteristics of breast cancer needs to be calculated in a multivariate regression analysis and verified in future multicenter and large-scale studies.

In conclusion, there were differences in the qualitative features and quantitative parameters of CEUS for breast cancer with different pathological grades and molecular subtypes. Contrast-enhanced ultrasound may be used to non-invasively predict the histological characteristics of breast cancer.

## Data availability statement

The original contributions presented in the study are included in the article/supplementary material. Further inquiries can be directed to the corresponding author.

## Ethics statement

The studies involving humans were approved by The Ethics Committee of the People’s Hospital of Guangxi Zhuang Autonomous Region. The studies were conducted in accordance with the local legislation and institutional requirements. The participants provided their written informed consent to participate in this study.

## Author contributions

L-LL: Data curation, Formal analysis, Writing – original draft, Validation. Q-LS: Writing – original draft, Data curation, Formal analysis, Validation. Y-XD: Data curation, Writing – original draft, Validation. W-WG: Methodology, Validation, Writing – original draft. H-ML: Validation, Formal analysis, Writing – review & editing. QH: Funding acquisition, Methodology, Project administration, Validation, Writing – original draft, Writing – review & editing.
